# Biomarcadores de Lesão Miocárdica e Complicações Cardíacas Associadas à Mortalidade em Pacientes com COVID-19

**DOI:** 10.36660/abc.20200372

**Published:** 2020-08-19

**Authors:** Paulo Ricardo Martins-Filho, José Augusto Soares Barreto-Filho, Victor Santana Santos

**Affiliations:** 1 Universidade Federal de Sergipe Aracaju SE Brasil Universidade Federal de Sergipe, Aracaju, SE - Brasil; 2 Fundação São Lucas Centro de Ensino e Pesquisa Aracaju SE Brasil Fundação São Lucas - Centro de Ensino e Pesquisa, Aracaju, SE - Brasil; 3 Universidade Federal de Alagoas Campus Arapiraca Arapiraca Al Brasil Universidade Federal de Alagoas – Campus Arapiraca, Arapiraca, Al – Brasil

**Keywords:** Coronavírus, COVID-19, SARS-CoV-2, Mortalidade

## Abstract

**Fundamento:**

O SARS-CoV-2 é um vírus de RNA emergente associado à doença respiratória aguda grave conhecida como COVID-19. Embora a COVID-19 seja predominantemente uma doença pulmonar, alguns pacientes apresentam graves danos cardiovasculares. Realizamos uma síntese de evidências quantitativas de dados clínicos, biomarcadores de lesão miocárdica e complicações cardíacas associadas ao óbito hospitalar em pacientes com COVID-19.

**Métodos:**

Buscamos nas bases de dados PubMed, Embase e Google Scholar para identificar estudos que comparassem dados clínicos, biomarcadores de lesão miocárdica e complicações cardíacas entre pacientes sobreviventes e não sobreviventes da COVID-19. Os tamanhos dos efeitos foram apresentados como diferença média ou diferença média padronizada para variáveis contínuas e razão de risco para variáveis dicotômicas, com intervalos de confiança de 95%. Foi utilizado um modelo de efeitos aleatórios para agrupar os resultados.

**Resultados:**

Foram incluídos seis estudos retrospectivos que relataram dados de 1.141 pacientes (832 sobreviventes e 309 não sobreviventes). Verificamos que condições cardiovasculares subjacentes; elevação de troponina cardíaca I de alta sensibilidade; N-terminal do pró-hormônio do peptídeo natriurético do tipo B e creatina quinase-MB; e complicações cardíacas foram associadas ao aumento do risco de óbito em pacientes com infecção por SARS-CoV-2.

**Conclusões:**

A confirmação de que condições cardiovasculares subjacentes, elevação de biomarcadores de lesão miocárdica durante a infecção por COVID-19 e descompensação cardiovascular aguda são preditores de mortalidade na infecção por SARS-CoV-2 deve incentivar novas pesquisas para esclarecer possíveis mecanismos e testar tratamentos adequados. (Arq Bras Cardiol. 2020; 115(2):273-277)

## Introdução

O coronavírus 2, da síndrome respiratória aguda grave 2 (SARS-CoV-2), é um novo coronavírus que causa uma doença infecciosa emergente com envolvimento pulmonar notável, conhecida como COVID-19. Além da hipótese de que pacientes cardíacos são mais suscetíveis à infecção por COVID-19 por desregulação dos receptores ACE2, relatos individuais preliminares têm demonstrado que pacientes com doença cardiovascular prévia apresentam maior risco de desfechos adversos. Além disso, pacientes que apresentam qualquer marcador clínico ou biológico de envolvimento cardíaco agudo durante a infecção por COVID-19 têm menor probabilidade de sobreviver.^1^

Embora o envolvimento cardíaco agudo, seja clínico ou revelado por biomarcadores, tenha sido descrito como uma condição comum entre pacientes hospitalizados por COVID-19, e esteja associado a um maior risco de óbito hospitalar,^2^ as evidências atualmente disponíveis são baseadas em estudos individuais com dados potencialmente sobrepostos.^3^ Portanto, uma síntese de evidências pode ajudar a confirmar esses achados. Neste estudo, realizamos uma síntese de evidências quantitativas de dados clínicos, biomarcadores de lesão miocárdica e complicações cardíacas associadas ao óbito hospitalar em pacientes com COVID-19.

## Métodos

Esta revisão sistemática e metanálise foram conduzidas de acordo com as diretrizes de Itens de Relatório Preferidos para Revisões Sistemáticas e Meta-Análises (PRISMA).^4^ Em vista da necessidade urgente para esta revisão, registro de PROSPERO não foi solicitado.

Buscamos nas bases de dados PubMed, Embase e Google Scholar identificar estudos que comparassem dados clínicos, biomarcadores de lesão miocárdica e complicações cardíacas entre pacientes sobreviventes e não sobreviventes da COVID-19. Incluímos apenas os estudos com dados clínicos que relataram, no mínimo, as concentrações de troponina cardíaca I de alta sensibilidade (TnI-as). Considerou-se que os pacientes apresentavam lesão miocárdica aguda quando os níveis séricos de TnI-as estavam acima do limite superior de referência (LSR) do percentil 99. Insuficiência cardíaca foi definida quando o nível sérico de N-terminal do pró-hormônio do peptídeo natriurético do tipo B (NT-proBNP) excedia a faixa normal, e na presença de sintomas associados, como dispneia, ortopneia e edema nos membros inferiores. A arritmia foi definida como taquicardia ventricular rápida, com duração superior a 30 segundos, induzindo instabilidade hemodinâmica e/ou fibrilação ventricular, e bradicardia clinicamente significativa na eletrocardiografia. Nós excluímos as publicações com relatos potencialmente sobrepostos baseados na coleção de dados e localização, bem como estudos nos quais a extração dos dados não foi possível. No caso de dados potencialmente sobrepostos, nós selecionamos o estudo com as informações mais completas.

Os relatos foram selecionados em duas etapas, triagem de títulos e resumos e subsequente obtenção e triagem dos artigos em texto completo. Foram realizadas as buscas entre 1º de janeiro de 2020 e 14 de abril de 2020, sem restrições de idioma. As listas de referências de todos os estudos e revisões elegíveis também foram avaliadas para identificar estudos adicionais para inclusão. Foram utilizados os seguintes termos de pesquisa: “COVID-19”, “SARS-CoV-2” e “coronavírus”. Todos os relatos de COVID-19, independentemente do tema cardiovascular, foram revisados.

Os dados das publicações foram extraídos por dois autores e verificados quanto à precisão. O nosso resultado de interesse foi o óbito hospitalar. Foram consideradas variáveis independentes os dados clínicos (idade, sexo e comorbidades existentes), biomarcadores de lesões miocárdicas (TnI-as, NT-proBNP e creatina quinase-MB [CK-MB]) e complicações cardíacas (lesão cardíaca aguda, insuficiência cardíaca e arritmias).

A Ferramenta de Avaliação de Qualidade de Estudos de Coorte e Transversais Observacionais dos Institutos Nacionais da Saúde dos EUA (https://www.nhlbi.nih.gov/health-topics/study-quality-assessment-tools) foi utilizada para avaliar a qualidade de cada estudo. Esta ferramenta é composta por 14 itens que avaliam a representatividade e seleção da amostra, a descrição e a mensuração da exposição, o acompanhamento dos participantes e o tratamento das variáveis confundidoras. Os achados foram discutidos qualitativamente. As discordâncias foram resolvidas por discussão.

Os tamanhos dos efeitos foram apresentados como diferença média (DM) ou diferença média padronizada (DMP) para variáveis contínuas e como razão de risco (RR) para variáveis dicotômicas, com intervalos de confiança (IC) de 95%. Para calcular DM e DMP, as médias e os desvios padrões (DP) dos biomarcadores de lesão miocárdica foram obtidos de cada estudo. Se as médias e os DP não foram diretamente relatados na publicação, foram utilizados métodos indiretos de extrair estimativas.^5 , 6^ Quando os dados não foram apresentados em tabelas ou no texto e não foi possível entrar em contato com os autores, os dados foram extraídos usando o software de digitalização de gráficos WebPlotDigitizer (disponível em http://arohatgi.info/WebPlotDigitizer). Nem todos os estudos relataram dados sobre todas as variáveis preditoras, e a análise agrupada foi estimada a partir dos dados disponíveis para cada variável.

Foi utilizado um modelo de efeitos aleatórios para agrupar os resultados, e p <0,05 bicaudal foi usado para determinar a significância. A classificação de Cohen foi utilizada para interpretar a magnitude do tamanho do efeito dos biomarcadores de lesão miocárdica. DMP > 0,8 foi considerada de grande tamanho de efeito. A heterogeneidade estatística foi quantificada pelo índice I^2^ , e potencial viés de publicação foi analisado para TnI-as, utilizando o teste de regressão de Egger e inspeção visual de gráficos de funil. Devido ao número pequeno de estudos que relataram dados para NT-proBNP e CK-MB, a análise de viés de publicação não foi realizada para estes. As análises foram realizadas utilizando Review Manager 5.3 (Cochrane IMS, Copenhague, Dinamarca).

## Resultados

Após a triagem de 8.091 títulos e resumos, 31 artigos foram avaliados em texto completo para elegibilidade. Foram excluídos 25 estudos, dos quais sete foram por causa de dados potencialmente sobrepostos. Foram incluídos seis estudos retrospectivos,^1 , 7 - 11^ fornecendo dados de 1.141 pacientes (633 de sexo masculino e 508 de sexo feminino), com infecção confirmada por SARS-CoV-2, 832 sobreviventes e 309 não sobreviventes. A Tabela 1 mostra os detalhes dos estudos incluídos.

**Tabela 1:** Características dos estudos incluídos e dados clínicos de pacientes com COVID-19, incluindo óbitos hospitalares

Autores	Desenho	Localização	Coleção de dados	Tamanho da amostra	Idade*	Sexo		Óbitos hospitalares	Sobreviventes

						Masculino	Feminino		
Zhou et al, 2020^1^	Coorte retrospectiva	Jinyintan Hospital and Wuhan Pulmonary Hospital	Dez 29, 2019 a Jan 31, 2020	191	56,3 (15,6)	119	72	54	137
Cao et al, 2020^7^	Coorte retrospectiva	Zhongnan Hospital of Wuhan University	Jan 3, 2020 a Fev 1, 2020	102	52,7 (22,2)	53	49	17	85
Chen et al, 2020^8^	Coorte retrospectiva	Tongji Medical College of Wuhan	Jan 13, 2020 a Fev 28, 2020	274	58,7 (19,3)	171	103	113	161
Guo et al, 2020^9^	Coorte retrospectiva	Seventh Hospital of Wuhan	Jan 23, 2020 a Fev 23, 2020	187	58,5 (14,7)	91	96	43	144
Wang et al, 2020^10^	Coorte retrospectiva	Renmin Hospital of Wuhan University	Jan 1, 2020 a Fev 6, 2020	339	70,0 (8,2)	166	173	65	274
Zhang et al, 2020^11^	Coorte retrospectiva	Wuhan No.1 Hospital	Dez 25, 2019 a Fev 15, 2020	48	70,6 (13,4)	33	15	17	31

*Dados relatados como média e desvio padrão.

O risco de viés dos estudos é mostrado na e-Tabela 1 no conteúdo digital suplementar. Todos os estudos tinham objetivos e critérios de elegibilidade claramente definidos, recrutaram sujeitos da mesma população e descreveram as definições dos fatores de exposição e dos desfechos. No entanto, os estudos não foram capazes de determinar se o tamanho da amostra era representativo da população. Além disso, nenhum dos estudos realizou análise para ajuste de fatores de confusão.

Os resultados da metanálise mostraram diferenças na idade entre os grupos. Não sobreviventes de COVID-19 eram mais velhos em comparação com os sobreviventes (DM = 14,3 anos, IC 95% 9,2 a 19,4). Sexo masculino (RR = 1,3, IC 95% 1,2 a 1,4), a presença de hipertensão existente (RR = 1,7, IC 95% 1,2 a 2,4) e doença cardiovascular (RR = 3,3, IC 95% 1,4 a 7,8) também foram associados ao aumento do risco de mortalidade.

A metanálise dos biomarcadores de lesão miocárdica demonstraram um grande aumento de TnI-as (DMP = 1,0, IC 95% 0,8 a 1,2), NT-proBNP (DMP = 1,1, IC 95% 0,7 a 1,4) e CK-MB (DMP = 1,0, IC 95% 0,2 a 1,8) em pacientes não sobreviventes. Valores elevados de TnI-as acima do LSR do percentil 99 foram associados a um aumento de 8 vezes no risco de óbito hospitalar (RR = 8,0, IC 95% 2,2 a 28,5). Não foram observadas evidências de vieses de publicação substancial para TnI-as. Foi verificado que as complicações cardíacas, incluindo lesão cardíaca aguda (RR = 8,9, IC 95% 4,2 a 19,3), insuficiência cardíaca (RR = 5,1, IC 95% 2,5 a 10,7) e arritmias (RR = 4,9, IC 95% 1,2 a 10,9) eram fatores de risco para óbito relacionado à COVID-19. Comparações de dados clínicos, biomarcadores da lesão miocárdica e complicações cardíacas entre não sobreviventes e sobreviventes da COVID-19 são exibidas na Tabela 2 . Os gráficos de floresta e os gráficos de funil são apresentados no conteúdo digital suplementar (e-Figuras 1 – 3).

**Tabela 2:** Comparação de dados clínicos, biomarcadores da lesão miocárdica e complicações cardíacas entre não sobreviventes e sobreviventes da COVID-19

Parâmetro	DM (IC 95%) entre sobreviventes e não sobreviventes	DMP (IC 95%) entre sobreviventes e não sobreviventes	RR (IC 95%)	Valor de p	I^2^
**Clínicos**					
Idade, anos	14,3 (9,2 a 19,4)	-	-	< 0,001	88%
Masculino	-	-	1,3 (1,2 a 1,4)	< 0,001	0%
Comorbidades					
Hipertensão	-	-	1,7 (1,2 a 2,4)	0,001	74%
Doença cardiovascular	-	-	3,3 (1,4 a 7,7)	0,005	70%
**Biomarcadores de lesão miocárdica**					
TnI-as	-	1,0 (0,8 a 1,2)	-	< 0,001	42%
TnI-as (> percentil 99)	-	-	8,0 (2,2 a 28,5)	0,001	93%
NT-proBNP	-	1,1 (0,7 a 1,4)	-	< 0,001	50%
CK-MB	-	1,0 (0,2 a 1,8)	-	0,010	81%
Complicações cardíacas					
Lesão cardíaca aguda	-	-	8,9 (4,2 a 19,1)	< 0,001	79%
Insuficiência cardíaca	-	-	5,1 (2,5 a 10,7)	< 0,001	75%
Arritmias	-	-	4,9 (1,2 a 19,0)	0,020	85%

*DM: diferença média; DMP: diferença média padronizada; RR: razão de risco; IC: intervalo de confiança. Resultados positivos para DMP indicam níveis aumentados de biomarcadores em pacientes não sobreviventes.*

## Discussão

A vigilância de eventos cardiovasculares associados à COVID-19 parece bem justificada.^12^ Este estudo confirma e quantifica melhor a associação de biomarcadores da lesão miocárdica e/ou complicações cardíacas agudas com o óbito hospitalar em pacientes com COVID-19. No entanto, ainda não está claro se o envolvimento cardíaco agudo é provocado principalmente pela SARS-CoV-2 ou se é um envolvimento cardíaco multifatorial inespecífico de uma infecção sistêmica grave.^13^ Tem sido proposto que o SARS-CoV-2 possa induzir a lesão cardíaca por múltiplos mecanismos, incluindo invasão viral direta de cardiomiócitos e subsequente miocardite, uma vez que partículas virais têm sido identificadas nas células do miocárdio.^14^ Porém, alterações na TnI ao longo do tempo e a ausência de sinais típicos na ecocardiografia e no ECG em pacientes com COVID-19 sugeriram que a lesão miocárdica em pacientes com COVID-19 esteja mais provavelmente relacionada às consequências sistêmicas da doença.^15^

Outros mecanismos plausíveis que têm sido sugeridos para explicar a elevação da troponina nesse cenário incluem infarto do miocárdio tipo 1 e, principalmente, tipo 2 devido à síndrome do desconforto respiratório agudo, sepse, tempestade de citocinas e até síndrome de Takotsubo.^16^ Portanto, a infecção por SARS-CoV-2 pode induzir novas lesões cardíacas e/ou atuar como um fator precipitante para agravar as doenças cardiovasculares subjacentes e levar ao óbito.

Nesta metanálise, analisamos biomarcadores bem estabelecidos para o diagnóstico de lesão miocárdica e a previsão de desfechos. A elevação de TnI-as, NT-proBNP e CK-MB foi associada ao aumento do risco de óbito em pacientes com infecção por SARS-CoV-2.

O manejo de pacientes com biomarcadores de lesão miocárdica e descompensação cardiovascular aguda é principalmente baseado em cuidados de suporte e abordagem individualizada para melhor orientar o tratamento. Infelizmente, não temos evidências para orientar o uso adequado de agentes antiplaquetários, anticoagulantes, betabloqueadores, inibidores da ECA e estatinas neste cenário crítico, e devemos adaptar o conhecimento atual.^17^ Por exemplo, foi recentemente sugerido que os inibidores do sistema renina-angiotensina-aldosterona possam ser deletérios ou benéficos para pacientes com COVID-19,^18^ mas ainda não temos as evidências definitivas para esta decisão.

Os achados deste estudo devem ser tratados com cautela. Suas limitações principais são as seguintes: (1) Os estudos são limitados a uma única região e isto reduziu a nossa capacidade de verificar possível variabilidade populacional; (2) houve heterogeneidade moderada a alta entre os estudos e (3) os estudos não realizaram análise para ajuste de fatores confundidores e seus resultados foram baseados em modelos univariados padrões.

## Conclusões

Esta metanálise confirma que as condições cardiovasculares subjacentes, a elevação dos biomarcadores da lesão miocárdica durante a infecção por COVID-19 e a descompensação cardiovascular aguda são preditores de mortalidade na infecção por SARS-CoV-2. Estudos futuros são necessários para esclarecer os possíveis mecanismos de lesão cardiovascular e testar tratamentos adequados.
