# Supplementary material for: Biomarcadores de Lesão Miocárdica e Complicações Cardíacas Associadas à Mortalidade em Pacientes com COVID-19
Source: Arq Bras Cardiol. 2020 Aug 19;115(2):273–7. [Article in Portuguese] doi: 10.36660/abc.20200372 (PMC8384270; doi:10.36660/abc.20200372)
Supplement: Translation

# Translation: Myocardial Injury Biomarkers and Cardiac Complications Associated with Mortality in Patients with COVID-19

**Authors:** Paulo Ricardo Martins-Filho, José Augusto Soares Barreto-Filho, Victor Santana Santos

**Affiliations:** 1 Brazil Universidade Federal de Sergipe, Aracaju, SE - Brazil; 2 Brazil Fundação São Lucas - Centro de Ensino e Pesquisa, Aracaju, SE - Brazil; 3 Brazil Universidade Federal de Alagoas – Campus Arapiraca, Arapiraca, Al – Brazil

**Keywords:** Coronavirus, COVID-19, SARS-CoV-2, Mortality

## Abstract

**Background:**

SARS-CoV-2 is an emerging RNA virus associated with a severe acute respiratory disease known as COVID-19. Although COVID-19 is predominantly a pulmonary disease, some patients have severe cardiovascular damage. We performed a quantitative evidence synthesis of clinical data, myocardial injury biomarkers, and cardiac complications associated with in-hospital death in patients with COVID-19.

**Methods:**

We searched the databases PubMed, Embase, and Google Scholar to identify studies comparing clinical data, myocardial injury biomarkers, and cardiac complications between non-survivors and survivors of COVID-19. Effect sizes were reported as mean difference or standardized mean difference for continuous variables and risk ratio for dichotomous variables with 95% confidence intervals. A random effects model was used to pool the results.

**Results:**

Six retrospective studies reporting data from 1,141 patients (832 survivors and 309 non-survivors) were included. We found that underlying cardiovascular conditions; elevation of high-sensitivity cardiac troponin I, N-terminal pro-B-type natriuretic peptide, and creatine kinase-MB; and cardiac complications were associated with increased risk of death for patients with SARS-CoV-2 infection.

**Conclusions:**

The confirmation that underlying cardiovascular conditions, elevation of myocardial injury biomarkers during COVID-19 infection, and acute cardiovascular decompensation are predictors for mortality in SARS-CoV-2 infection must encourage new research to clarify potential mechanisms and test appropriate treatments. (Arq Bras Cardiol. 2020; 115(2):273-277)

## Introduction

Severe acute respiratory syndrome coronavirus 2 (SARS-CoV-2) is a novel coronavirus that leads to an emerging infectious disease with remarkable pulmonary involvement, known as COVID-19. In addition to the hypothesis that cardiac patients are more susceptible to COVID-19 infection via ACE2 receptor dysregulation, preliminary individual reports have shown that patients with previous cardiovascular disease are at a higher risk of adverse outcomes. Moreover, patients who present any clinical or biological marker of acute cardiac involvement during COVID-19 infection are less likely to survive.^1^

Although acute cardiac involvement, whether clinical or revealed by biomarkers, has been described as a common condition among patients hospitalized with COVID-19, and it is associated with a higher risk of in-hospital death,^2^ current available evidence is based on individual studies with potentially overlapping data.^3^ Therefore, a synthesis of evidence can help confirm these findings. In this study, we performed a quantitative evidence synthesis of clinical data, myocardial injury biomarkers, and cardiac complications associated with in-hospital death in patients with COVID-19.

## Methods

This systematic review and meta-analysis was conducted in accordance with the Preferred Reporting Items for Systematic Reviews and Meta-Analyses (PRISMA) guidelines.^4^ Given the urgent need for this review, PROSPERO registration was not sought.

We searched the databases PubMed, Embase, and Google Scholar to identify studies comparing clinical data, myocardial injury biomarkers, and cardiac complications between non-survivors and survivors of COVID-19. We included only studies with clinical data that provided, at least, concentrations of high-sensitivity cardiac troponin I (hs-cTnI). Patients were considered to have acute myocardial injury if serum levels of hs-cTnI were above the 99^th^percentile upper reference limit (URL). Heart failure was defined when the serum level of N-terminal pro-B-type natriuretic peptide (NT-proBNP) exceeded the normal range and in the presence of associated symptoms, such as dyspnea, orthopnea, and lower extremity edema. Arrhythmia was defined as rapid ventricular tachycardia lasting more than 30 seconds, inducing hemodynamic instability and/or ventricular fibrillation, and clinically significant bradycardia on electrocardiography. We excluded publications with potentially overlapping reports based on data collection and setting and studies from which data extraction was not possible. In the event of potentially overlapping data, we selected the study with the most complete information.

Reports were screened in two stages, screening of titles and abstracts followed by the retrieval and screening of full-text articles. Searches were performed from January 1, 2020 to April 14, 2020, without language restrictions. The reference lists of all eligible studies and reviews were also evaluated to identify additional studies for inclusion. The following search terms were used: “COVID-19”, “SARS-CoV-2”, and “coronavirus”. All COVID-19 reports, irrespective of cardiovascular topic, were reviewed.

Data from publications were extracted by two authors and crosschecked for accuracy. Our outcome of interest was in-hospital death. Clinical data (age, sex, and existing comorbidities), myocardial injury biomarkers (hs-cTnI, NT-proBNP, and creatine kinase-MB [CK-MB]), and cardiac complications (acute cardiac injury, heart failure, and arrhythmias) were considered independent variables.

The Quality Assessment Tool for Observational Cohort and Cross-Sectional Studies of the National Institutes of Health (https://www.nhlbi.nih.gov/health-topics/study-quality-assessment-tools) was used to grade the quality of each study. This tool is composed of 14 items that evaluate the representativeness and selection of the sample, description and measurement of exposure, follow-up of participants, and treatment of confounding factors. The findings were discussed qualitatively. Disagreements were resolved by discussion.

Effect sizes were reported as mean difference (MD) or standardized mean difference (SMD) for continuous variables and risk ratio (RR) for dichotomous variables with 95% confidence intervals (CI). To calculate MD and SMD, means and standard deviations (SD) of myocardial injury biomarkers were obtained for each study. If the means and SD were not directly reported in the publication, indirect methods of extracting estimates were used.^5 , 6^ When data were not presented in tables or in the text and the authors could not be reached, data were extracted using WebPlotDigitizer graph digitization software (available at http://arohatgi.info/WebPlotDigitizer). Not all studies reported data on all predictor variables, and the pooled analysis was estimated from the data available for each variable.

A random effects model was used to pool the results, and 2-tailed p < 0.05 was used to determine significance. Cohen’s classification was used to interpret magnitude of the effect size for myocardial injury biomarkers. SMD > 0.8 was considered a large effect size. Statistical heterogeneity was quantified by the I^2^ index, and potential for publication bias was analyzed for hs-cTnI using Egger’s regression test and visual inspection of funnel plots. Because of the small number of studies reporting data for NT-proBNP and CK-MB, analysis of publication bias was not performed. Analyses were conducted using Review Manager 5.3 (Cochrane IMS, Copenhagen, Denmark).

## Results

After screening 8,091 titles and abstracts, 31 full-text articles were assessed for eligibility and 25 studies were excluded, seven of which were due to potentially overlapping data. Six retrospective studies^1 , 7 - 11^ were included, providing data from 1,141 patients (633 male and 508 female) with confirmed SARS-CoV-2 infection, 832 survivors and 309 non-survivors. Details of included studies are shown in Table 1 .

**Table 1:** Characteristics of included studies and clinical data of patients with COVID-19, including in-hospital deaths

Authors	Design	Setting	Data collection	Sample size	Age*	Sex		In-hospital deaths	Survivors

						Male	Female		
Zhou et al, 2020^1^	Retrospective cohort	Jinyintan Hospital and Wuhan Pulmonary Hospital	Dec 29, 2019 to Jan 31, 2020	191	56.3 (15.6)	119	72	54	137
Cao et al, 2020^7^	Retrospective cohort	Zhongnan Hospital of Wuhan University	Jan 3, 2020 to Feb 1, 2020	102	52.7 (22.2)	53	49	17	85
Chen et al, 2020^8^	Retrospective cohort	Tongji Medical College of Wuhan	Jan 13, 2020 to Feb 28, 2020	274	58.7 (19.3)	171	103	113	161
Guo et al, 2020^9^	Retrospective cohort	Seventh Hospital of Wuhan	Jan 23, 2020 to Feb 23, 2020	187	58.5 (14.7)	91	96	43	144
Wang et al, 2020^10^	Retrospective cohort	Renmin Hospital of Wuhan University	Jan 1, 2020 to Feb 6, 2020	339	70.0 (8.2)	166	173	65	274
Zhang et al, 2020^11^	Retrospective cohort	Wuhan No.1 Hospital	Dec 25, 2019 to Feb 15, 2020	48	70.6 (13.4)	33	15	17	31

*Data reported in mean and standard deviation.

The risk of bias of the studies is showed in e-Table 1 in the supplemental digital content. All studies had clear objectives and eligibility criteria, recruited subjects from the same population, and described the definitions of exposure factors and outcomes. However, studies have not been able to determine whether the sample size was representative for the population. In addition, none of the studies performed analysis for adjustment of confounding factors.

Results of meta-analysis showed differences in age between groups. Non-survivors of COVID-19 were older compared to survivors (MD = 14.3 years, 95% CI 9.2 to 19.4). Male sex (RR = 1.3, 95% CI 1.2 to 1.4), the presence of existing hypertension (RR = 1.7, 95% CI 1.2 to 2.4), and cardiovascular disease (RR = 3.3, 95% CI 1.4 to 7.8) were also associated with increased risk of mortality.

The meta-analysis of myocardial injury biomarkers showed a large increase in hs-cTnI (SMD = 1.0, 95% CI 0.8 to 1.2), NT-proBNP (SMD = 1.1, 95% CI 0.7 to 1.4), and CK-MB (SMD = 1.0, 95% CI 0.2 to 1.8) in non-survivor patients. Elevated hs-cTnI values above the 99^th^percentile URL were associated with 8-fold increase in the risk of in-hospital death (RR = 8.0, 95% CI 2.2 to 28.5). No evidence of substantial publication bias was observed for hs-cTnI. Cardiac complications, including acute cardiac injury (RR = 8.9, 95% CI 4.2 to 19.3), heart failure (RR = 5.1, 95% CI 2.5 to 10.7), and arrhythmias (RR= 4.9, 95% CI 1.2 to 10.9) were found to be risk factors for COVID-19 related death. Comparisons of clinical data, myocardial injury biomarkers, and cardiac complications between non-survivors and survivors of COVID-19 are displayed in Table 2 . Forest plots and funnel plots are shown in the supplemental digital content (e-Figures 1 – 3).

**Table 2:** Comparison of clinical data, myocardial injury biomarkers, and cardiac complications between non-survivors and survivors of COVID-19

Parameter	MD (95% CI) between non-survivors and survivors	SMD (95% CI) between non-survivors and survivors	RR (95% CI)	p value	I^2^
**Clinical**					
Age, years	14.3 (9.2 to 19.4)	-	-	< 0.001	88%
Male	-	-	1.3 (1.2 to 1.4)	< 0.001	0%
Comorbidities					
Hypertension	-	-	1.7 (1.2 to 2.4)	0.001	74%
Cardiovascular disease	-	-	3.3 (1.4 to 7.7)	0.005	70%
**Myocardial injury biomarkers**					
hs-cTnI	-	1.0 (0.8 to 1.2)	-	< 0.001	42%
hs-cTnI (> 99th percentile)	-	-	8.0 (2.2 to 28.5)	0.001	93%
NT-proBNP	-	1.1 (0.7 to 1.4)	-	< 0.001	50%
CK-MB	-	1.0 (0.2 to 1.8)	-	0.010	81%
**Cardiac complications**					
Acute cardiac injury	-	-	8.9 (4.2 to 19.1)	< 0.001	79%
Heart failure	-	-	5.1 (2.5 to 10.7)	< 0.001	75%
Arrhythmias	-	-	4.9 (1.2 to 19.0)	0.020	85%

*MD: mean difference; SMD: standardized mean difference; RR: risk ratio; CI: confidence interval. Positive results for SMD indicate increased levels of biomarkers in non-survivor patients.*

## Discussion

Surveillance of cardiovascular events associated with COVID-19 seems very warranted.^12^ This study confirms and better quantifies the association between myocardial injury biomarkers and/or acute cardiac complications with in-hospital death in patients with COVID-19. However, it remains unclear whether acute cardiac involvement is primarily provoked by SARS-CoV-2 or whether it is a multifactorial non-specific cardiac involvement of a severe systemic infection.^13^ It has been proposed that SARS-CoV-2 may lead to cardiac injury via multiple mechanisms including direct viral invasion of cardiomyocytes and subsequent myocarditis, since viral particles have been identified in myocardial cells.^14^ However, changes in cTnI over time and the absence of typical signs on echocardiography and ECG in patients with COVID-19 have suggested that myocardial injury in patients with COVID-19 is more likely related to systemic consequences of disease.^15^

Other plausible mechanisms that have been suggested to explain troponin elevation in this scenario include type 1 and, mainly, type 2 myocardial infarction due to acute respiratory distress syndrome, sepsis, cytokine storm, and even Takotsubo syndrome.^16^ Therefore, SARS-CoV-2 infection may either induce new cardiac injuries and/or act as a precipitating factor to worsen underlying cardiovascular diseases and lead to death.

In this meta-analysis, we analyzed well-established biomarkers for myocardial injury diagnosis and outcome prediction. Elevation of hs-cTnI, NT-proBNP, and CK-MB were associated with increased risk of death in patients with SARS-CoV-2 infection.

Management of patients with myocardial injury biomarkers and acute cardiovascular decompensation is primarily based on supportive care and individualized approach to better guide treatment. Unfortunately, we do not have evidence to guide the proper use of antiplatelet agents, anticoagulants, β-blockers, ACE inhibitors, and statins in this critical scenario, and we must adapt the current knowledge.^17^ For instance, it has recently been suggested that renin-angiotensin-aldosterone system inhibitors could be deleterious or beneficial for patients with COVID-19,^18^ but we lack definitive evidence for this decision.

The findings of this study should be treated with caution. Its main limitations include the following: (1) Studies are limited to a single region and this has reduced our ability to verify possible population variability; (2) there was a moderate to high between-study heterogeneity, and (3) studies did not perform analysis for adjustment of confounding factors and their results were based on standard univariate models.

## Conclusions

This meta-analysis confirms that underlying cardiovascular conditions, elevation of myocardial injury biomarkers during COVID-19 infection, and acute cardiovascular decompensation are predictors for mortality in SARS-CoV-2 infection. Further studies are needed to clarify potential mechanisms of cardiovascular injury and test appropriate treatments.
